# Management of Diabetes in Candidates for Liver Transplantation and in Transplant Recipients

**DOI:** 10.1097/TP.0000000000003867

**Published:** 2021-06-23

**Authors:** Lucia Brodosi, Salvatore Petta, Maria L. Petroni, Giulio Marchesini, Maria C. Morelli

**Affiliations:** 1 IRCCS – Azienda Ospedaliero-Universitaria di Bologna, Bologna, Italy.; 2 Department of Medical and Surgical Sciences, Alma Mater University, Bologna, Italy.; 3 Section of Gastroenterology and Hepatology, PROMISE, University of Palermo, Palermo, Italy.

## Abstract

Diabetes is common in patients waitlisted for liver transplantation because of end-stage liver disease or hepatocellular cancer as well as in posttransplant phase (posttransplantation diabetes mellitus). In both conditions, the presence of diabetes severely affects disease burden and long-term clinical outcomes; careful monitoring and appropriate treatment are pivotal to reduce cardiovascular events and graft and recipients’ death. We thoroughly reviewed the epidemiology of diabetes in the transplant setting and the different therapeutic options, from lifestyle intervention to antidiabetic drug use—including the most recent drug classes available—and to the inclusion of bariatric surgery in the treatment cascade. In waitlisted patients, the old paradigm that insulin should be the treatment of choice in the presence of severe liver dysfunction is no longer valid; novel antidiabetic agents may provide adequate glucose control without the risk of hypoglycemia, also offering cardiovascular protection. The same evidence applies to the posttransplant phase, where oral or injectable noninsulin agents should be considered to treat patients to target, limiting the impact of disease on daily living, without interaction with immunosuppressive regimens. The increasing prevalence of liver disease of metabolic origin (nonalcoholic fatty liver) among liver transplant candidates, also having a higher risk of noncirrhotic hepatocellular cancer, is likely to accelerate the acceptance of new drugs and invasive procedures, as suggested by international guidelines. Intensive lifestyle intervention programs remain however mandatory, both before and after transplantation. Achievement of adequate control is mandatory to increase candidacy, to prevent delisting, and to improve long-term outcomes.

## INTRODUCTION

Liver transplantation (LTx) is the only curative treatment for acute and chronic end-stage liver diseases (ESLD), as well as for unresectable primary liver cancer. Diabetes mellitus, namely type 2 diabetes (T2D), is recognized as one of the most common and severe complications of ESLD. Both before and after solid organ transplantation, T2D favors infections, cardiovascular (CV) events and end-stage renal disease that may preclude waitlisting, as well as reduce organ and patient survival after LTx.^1^ The prevalence of diabetes depends on several factors, including chance association in the present epidemics of obesity, the importance of the liver in the regulation of glucose metabolism (the so-called hepatogenous diabetes), and finally specific conditions, fueling the development of posttransplantation diabetes mellitus (PTDM). The term PTDM was definitely adopted in a 2013 international conference defining newly diagnosed diabetes in the post-LTx setting, regardless of timing or whether it was present but undiagnosed before transplant.^2^

The present review focuses on problems derived from the presence of diabetes, from diagnosis to desired targets and monitoring in LTx candidates (Table 1),^3-5^ as well as on the challenges in post-LTx diabetes management.

**TABLE 1.** Diagnosis, glycemic targets, and monitoring of diabetes, with reference to problems in transplantation settings

**Measure**	**Fasting plasma glucose**	**2-h glucose (OGTT**)^*a*^	**Random glucose**	**Glycosylated hemoglobin (HbA1c**)^*b*^
Diagnosis				
Diabetes	≥7 mmol/L^*c*^(≥126 mg/dL)	≥11 mmol/L^*c*^(≥200 mg/dL)	≥11 mmol/L^*c,d*^(≥200 mg/dL)	≥48 mmol/mol^*c*^ (6.5%)
Prediabetes	5.6–6.9 mmol/L (100–125 mg/dL)	7.8–11 mmol/L (140–199 mg/dL)	—	39–47 mmol/mol(5.7%–6.4%)
Glycemic targets^*c*^	—	—	5.0–8.3 mmol/L^*e*^ (90–150 mg/dL)	<53 mmol/mol(7%)
Monitoring	At any control visit	Consider to repeat according to changes in general state	Measured at fixed daily time points in insulin-treated patients	3- to 6-mo interval

Diagnostic criteria are dictated by guidelines and do not differ between pre- and post-LTx diabetes (PTDM); targets and monitoring are dictated by frailty.

^*a*^A 2-h OGTT (75 g glucose in 200 mL water) is recommended in subjects with overweight/obesity, hypertension, dyslipidemia, and in the presence of a family history (first-degree relatives).^3^

^*b*^Glycated hemoglobin is formed in the blood by nonenzymatic glycosylation; whenever hemoglobin values are higher or lower than normal, HbA1c values can overestimate or underestimate glycemic control. These conditions include sickle cell disease and other hemoglobin variants, hemodialysis, recent blood loss or transfusion, erythropoietin therapy, and iron-dependent anemia.^5^

^*c*^Two values exceeding the cutoff are required for the diagnosis.

^*d*^In the presence of typical symptoms of diabetes: polyuria, polydipsia, and weight loss.

^*e*^Glycemic targets may be more or less stringent in relation to the risks associated with hypoglycemia (also dependent on antidiabetic drug use), diabetes duration, life expectancy, comorbidities (in particular, vascular complications), patients’ motivation, resources, and support system (caregivers).^4^

HbA1c, A1c glycated hemoglobin; LTx, liver transplantation; OGTT, oral glucose tolerance test; PTDM, posttransplantation diabetes mellitus.

### Diabetes and Liver Transplantation—Literature Search

A comprehensive search for articles, guidelines, and consensus reports was performed using specific words/MeSH terms (Liver transplantation, diabetes, outcome) and filters (adult, +19 y) for the years 2000–2020. Only 3 guidelines on adult LTx, with focus on diabetes and related metabolic diseases, were retrieved (Table 2).^6-9^ One consensus report was specifically devoted to nonalcoholic steatohepatitis (NASH)^10^ and summarized the evidence reviewed during a meeting organized by the International Liver Transplantation Society in 2018.^11^ Two documents^7,10^ were scored following the grade system.^12^ A specific document for the management of PTDM,^13^ published in 2005, was not considered because of updates by the 2013 Consensus.^2^

**TABLE 2.** Guidelines and International Consensus documents on liver transplantation, with specific reference to the importance of diabetes assessment, definition, and management

**Area**	**AGA Practice Guideline** ^6^	**EASL Clinical Guidelines** ^7^	**UK Clinical Guideline** ^8,9^	**ILTS Consensus (NASH**)^10,11^
Pre-LTx period				
Diagnosis	Undefined (ADA criteria)	Undefined	Undefined	Undefined
Importance of screening	Attention to CV risk in the presence of DM and other metabolic diseases	Extensive workup before registration on the waiting list, as obesity, hypertension, DM, and dyslipidemia increase morbidity	DM not included in the pre-LTx assessment (included obesity and MetSyn)Transplant Benefit Score (with DM among criteria for organ allocation) recently agreed in UK	Appropriate screening for hypertension, DM, and dyslipidemia highly recommended in NASH patients with indication for LTxDM at time of LTx (not obesity) is independent predictive factor for poorer post-LTx survival
Management	Managed with insulin and oral hypoglycemics. To be used with caution because of the risk of hypoglycemia	Pre-LTx DM and dyslipidemia should be managed as in the general population	—	Medical optimization of DM and other metabolic comorbidities strongly recommendedPioglitazone improves NASH features (except for fibrosis) and achieves NASH resolutionBariatric first or LTx-sleeve gastrectomy combined approach reasonable to manage modifiable risk factors and improve outcomesPhysical activity safe and feasible in patients awaiting LTx
Post-LTx period				
Risk factors	PTDM largely fueled by pre-LTx NASH indication	—	Risk factors for PTDM include HCV infection, male gender, corticosteroid use, pre-LTx DM, and CNIs (tacrolimus > ciclosporin)Post-LTx DM increases NASH recurrence	DM occurrence is exacerbated by steroid useSteatotic donor livers (mainly if steatosis >60%) are associated with poor graft function due to ischemia-reperfusion injuryDM associated with risk of post-LTx renal dysfunction
Treatment	—	Treatment of modifiable risk factors (arterial hypertension, hyperlipidemia, DM, and obesity) in the form of lifestyle changes and drug therapy be initiated as soon as possible to control metabolic risk factorsAvoid excessive weight gainHealthy diet and regular exercise programs represent effective management options	Advice and weight programs to decrease obesity riskEnsure close control with regular evaluationNo particular advantage for 1 particular antidiabetic drugRapid tapering of steroidsBenefit of targeted exercise/nutritional programs post-LTx are emerging but as yet unproven on solid outcomes	Treatment of comorbidities not different from other causesPTDM associated with poorer outcomeMost studies on treatment outcomes are limited by a lack of diabetes-specific dataNo solid data on outcomes comparing immediate LTx or optimal control of obesity and diabetes before LTx

No documents address the issue of DM in the immediate peritransplant period.

ADA, American Diabetes Association; AGA, American Gastroenterological Association; CNI, calcineurin inhibitor; CV, cardiovascular; DM, diabetes mellitus; EASL, European Association for the Study of the Liver; HCV, hepatitis C virus; ILTS, International Liver Transplantation Society; LTx, liver transplantation; MetSyn, metabolic syndrome; NASH, nonalcoholic steatohepatitis; PTDM, posttransplantation diabetes mellitus; UK, United Kingdom.

All guidelines suggest specific attention to diabetes in LTx candidates and in LTx recipients because of its high prevalence, particularly in NASH cases, and the impact of diabetes on complications and death in waitlisted patients and poorer outcomes after LTx. However, very few specific recommendations could be retrieved, except for strong recommendations for accurate CV function tests in the pre- and post-LTx period, for attention to the pending risk of renal dysfunction, and for the need for glucose control without risks of hypoglycemia.

### Prevalence of and Risk Factors for Pretransplantation Diabetes

**FIGURE 1.**
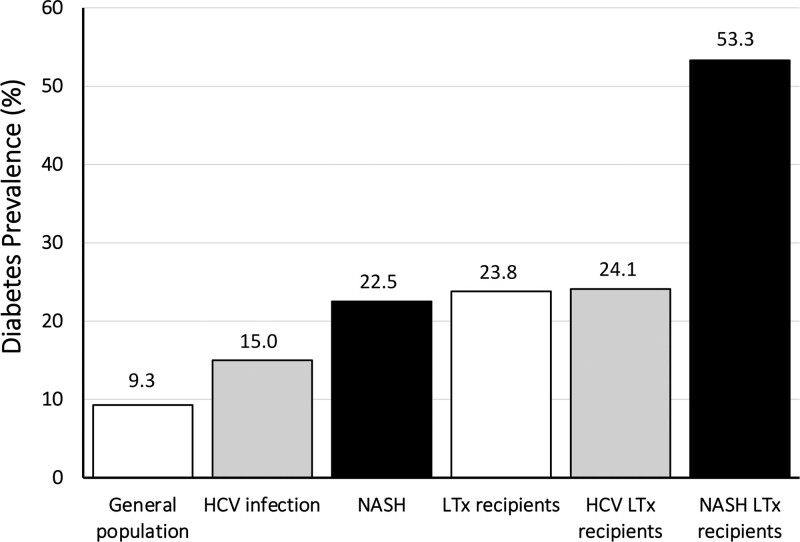
Prevalence of type 2 diabetes in the general population and in patients with chronic liver disease or LTx recipients, stratified by cause. Plotted from data retrieved from: general population,^14^ HCV infection,^15^ NASH,^16^ LTx recipients,^17^ and HCV and NASH LTx recipients.^18^ Note that data in LTx recipients are largely dependent on the relative proportion of patients with HCV and NASH cause. HCV, hepatitis C virus; LTx, liver transplantation; NASH, nonalcoholic steatohepatitis.

**FIGURE 2.**
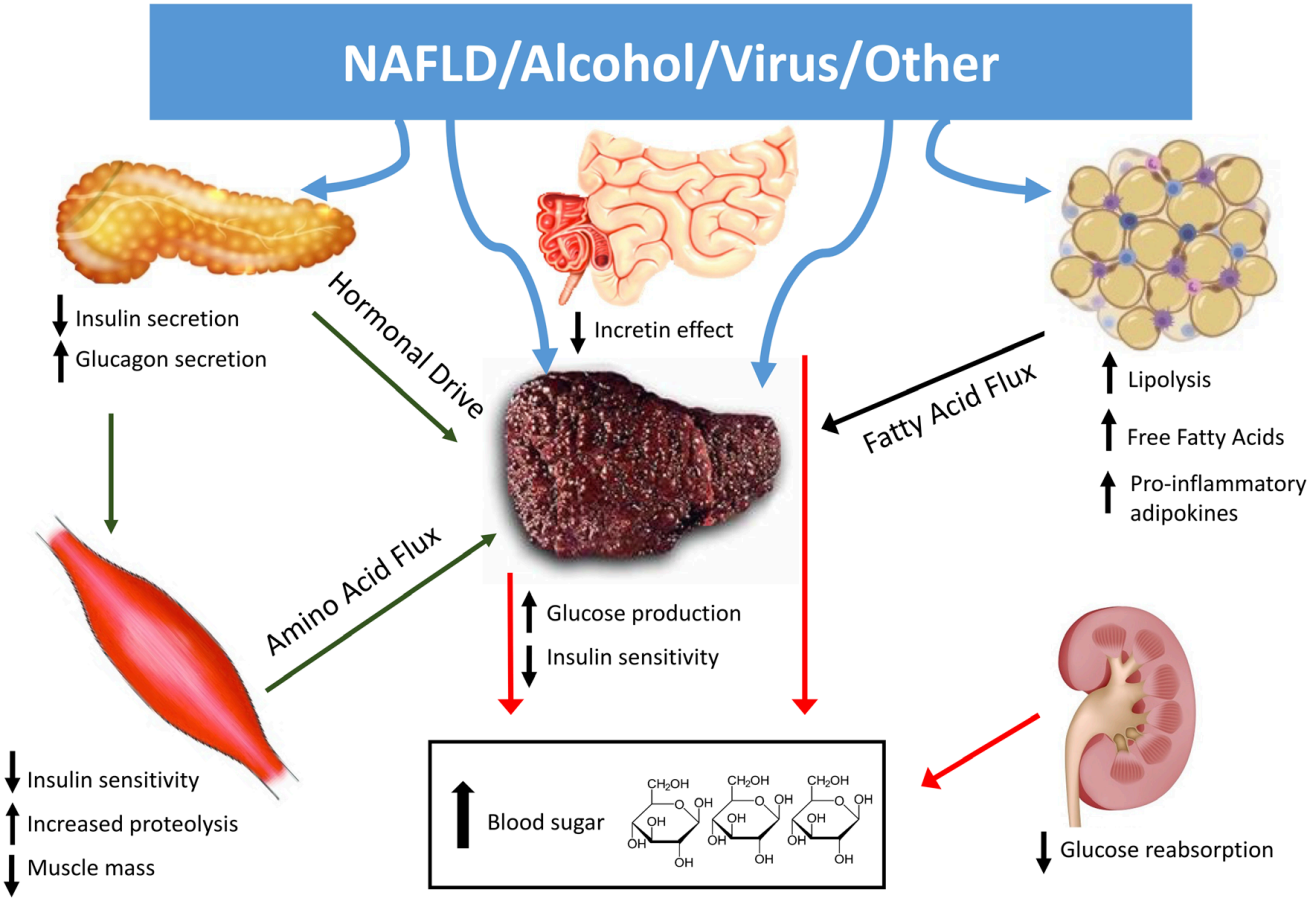
Pathogenic mechanisms responsible for diabetes in cirrhosis, targets of different antidiabetic agents. The presence of cirrhosis, with NAFLD, alcohol, and hepatitis C virus as more common causes, produces important changes in whole-body metabolism, involving other tissues and organs. Muscle and adipose tissue insulin resistance, aggravated by decreased muscle mass (sarcopenia), high glucagon, and defective insulin secretion by pancreatic islet cells, generates an accelerated flux of substrate to the liver resulting in increased hepatic glucose production. A defective incretin effect by small bowel cells and increased glucose reabsorption by the kidney further raise circulating glucose. In this context, incretins (GLP-1 receptor agonists and, less efficiently, DPP-4 inhibitors) and SGLT-2 inhibitors, by reducing glucose levels in a glucose-dependent manner, also lower whole-body insulin resistance. The activity of GLP-1 receptor agonists is magnified by extra-pancreatic effects (retarded gastric empty and neurotransmitter-mediated central anorexia). DPP-4, dipeptidyl peptidase-4; GLP-1, glucagon-like peptide-1; NAFLD, nonalcoholic fatty liver disease; SGLT-2, sodium-glucose cotransporter-2.

Globally, the prevalence of T2D is high and rising across the world. This picture is driven by aging and obesity; for the year 2019, the International Diabetes Federation reported a 9.3% worldwide prevalence for adults aged 20–79 y, estimated to increase to 10.2% and 10.9% in 2030 and 2045, respectively.^14^ The rate is much higher in chronic liver diseases (15% in hepatitis C virus [HCV] infection^15^ and 55.5% in nonalcoholic fatty liver disease [NAFLD])^16^ (Figure 1), translating into higher prevalence in LTx recipients (22.8%).^17^ In a systematic review, diabetes occurred in 49.0%–72.9% of NAFLD, in 24.0%–24.5% of HCV, and in 11.0%–52.0% of alcohol-related cases^18^ and was higher in NASH recipients (58.5%) versus non-NASH recipients (25.4%).^19^ Several pathogenic mechanisms can be advocated, from a chance association to the impaired insulin sensitivity/secretion related to specific causes, particularly the multiple metabolic triggers of NAFLD or HCV (Figure 2).^20,21^ Also, sarcopenia is likely to reduce glucose handling, favoring diabetes in individuals with decompensated cirrhosis or hepatocellular carcinoma (HCC). “Hepatogenous” diabetes occurs well before the development of cirrhosis, is proportional to the severity of liver dysfunction,^22^ and is potentially reversible following LTx.^23^

The above data provide a gloomy picture of the impact of diabetes in waitlisted patients and in LTx recipients. Because of the pandemic of diabesity,^24^ NAFLD is an emerging cause of chronic liver disease and its complications, including HCC—also observed in the precirrhotic stages^25^—and of liver decompensation, finally indicating LTx. European and United States transplant registries indicate NAFLD as the fastest growing indication for LTx in the United States and probably worldwide.^26^ In the United States, 21.7% of adult LTx Scientific Registry of Transplant Recipient candidates in 2002–2019 had NASH. Notably, the proportion of waitlisted non-HCC NAFLD increased about 5 times (from near 5% to near 25%) and that of HCC NAFLD about 24 times (from <1% to nearly 23%) in the period 2002–2018.^26^ In Europe, only 2.8% of adult LTx were performed for NAFLD-related disease between January 2007 and June 2017,^27^ but these figures are probably underestimated because of scarce awareness of NAFLD diagnostic criteria.

### Treatment of Diabetes in Waitlisted Candidates

Diabetes treatment in LTx candidates does not differ from the treatment of any patients with diabetes unless decompensated cirrhosis is present, particularly when complicated by progressive renal failure. Notably, the severity of renal involvement may be underestimated by common algorithms measuring glomerular filtration rate (eGFR),^28^ considering low sense of hunger and reduced food intake, low protein diet, sarcopenia, reduced creatinine synthesis, and dosage interference by bilirubin.^29^

The goal of diabetes treatment has completely changed in the past 15 y, moving from standard values of A1c glycosylated hemoglobin (HbA1c) (usually 7%, ie, 53 mmol/mol) to targets defined according to patient frailty.^30^ New drug classes have been approved, addressing the key factors responsible for the pathogenesis of diabetes (Figure 2), providing accurate glucose control coupled with protection on the CV system, reducing the risk of heart failure and progressive renal disease, and finally being safer in terms of hypoglycemia. A totally new flowchart has been set by international associations, and the new schedule is progressively being accepted in clinical practice.^30^ Excluding the very few patients with type 1 diabetes, where intensified insulin treatment remains the only possible option, the old criterion that insulin should be the treatment of choice for patients with cirrhosis has been progressively challenged by evidence that old and new antidiabetic drugs are safely used outside decompensated cirrhosis. In comparison with sulfonylureas and glinides, the novel classes of antidiabetic drugs provide glucose control without risk of hypoglycemia (Table 3), thus improving both life expectancy and quality of life, and should be the treatment of choice also in waitlisted patients, without forgetting weight-loss strategies, whenever needed.

**TABLE 3.** Glucose-lowering drugs for use in waitlisted patients with diabetes and following liver transplantation^*a*^

**Drug class**	**Molecule**	**Administration route/dosage**	**Expected beneficial effects**	**Possible adverse events**	**Biochemistry/areas of attention**	**Possible strategy to limit risks**
Biguanides	Metformin	Oral2000–2500 mg/d	↓ 0.5% HbA1cSlight weight reductionReduced risk of HE and HCC	DiarrheaNauseaRisk of lactic acidosis	Creatinine/eGFR and adjust the dose accordinglyStop the drug 48 h before iodinate contrast media injection	Start with half dosage and up-titrate or use slow-release products to prevent GI AEs and diarrhea
Glitazones	Pioglitazone^*b*^	Oral30–45 mg/d30–45 mg/d	↓ 0.5%–0.8% HbA1cReduced incidence of HCCFibrosis regression in NASH↓ CHD and cerebrovascular disease risk	Fluid retentionWeight gainNonosteoporotic fractures	AminotransferasesMonitor for ascites or heart failure development	Intensify lifestyle intervention to limit weight gain
α-glucosidase inhibitors	Acarbose	Oral150–300 mg/d	↓ 0.2%–0.5% HbA1cImproved ammonia and latent HE	DiarrheaFlatulence		Start with half dosage and titrate to prevent GI AEs
DPP-4 inhibitors	Sitagliptin^*b*^Vildagliptin^*b*^Alogliptin^*b*^Saxagliptin^*b*,*c*^Linagliptin^*b*,*c*^	Oral25–100 mg/dOral50–100 mg/dOral6.5–25 mg/dOral2.5–5 mg/dOral5 mg/d	↓ 0.5%–0.8% HbA1c		Creatinine/eGFR and adjust the dose according CKD stage (up to dialysis)No dose adjustment required for linagliptin	
GLP-1 receptor agonists^*d*^	DulaglutideExenatide LARLiraglutideLixisenatideSemaglutide	S.C./weekly 0.75–1.5S.C./weekly2 mgS.C./daily1.2–1.8S.C./daily10–20S.C./weekly0.5–1 mg	↓ 0.8%–1.0% HbA1cUsually 3%–5% weight loss (but much higher targets may be achieved, particularly with semaglutide)CVD and CKD protection	Nausea and decreased appetiteConstipationpossible risk of pancreatitisModest increase in heart rate	Amylase/lipaseheart rateHVPG in patients under β-blocking agents	Titrate to prevent GI AEsSmall, frequent and lipid-restricted meals to prevent GI AEs
SGLT-2 inhibitors	Canagliflozin^*b*^Dapagliflozin^*b*^Empagliflozin^*b*^Ertugliflozin^*b*^	Oral100–300 mg/dOral10 mg/dOral25–30 mg/dOral5–15 mg/d	↓ 0.5%–0.8% HbA1cUsually 2%–4% weight lossDiuretic effect similar to loopCVD, HF, and renal protection	Genital infectionsRisk of normoglycemic ketoacidosis	Electrolytes, creatinine, eGFR, albuminuriaDose adjustment according to eGFR (the lower the eGFR, the lower the effect of drug)	Recommend personal hygieneTitrate to evaluate tolerabilityEvaluate β-cell function to minimize the risk of ketoacidosis

^*a*^Note that in principle, no differences exist between pre- and post-LTx phase, and drug choice depends on patients’ frailty.

^*b*^Available in preestablished combination with metformin.

^*c*^Available in preestablished combination with SGLT-2 inhibitors.

^*d*^Albiglutide, another weekly dosing, albumin-bound GLP-1RA was removed from the market in 2018 because of limited prescribing and warning for risk of hypersensitivity reactions.

AE, adverse event; CHD, coronary heart disease; CKD, chronic kidney disease; CVD, cardiovascular disease; DPP-4, dipeptidyl peptidase-4; eGFR, estimated glomerular filtration rate; GI, gastrointestinal; GLP-1, glucagon-like peptide-1; GLP-1RA, GLP-1 receptor agonist; HbA1c, A1c glycated hemoglobin; HCC, hepatocellular carcinoma; HE, hepatic encephalopathy; HF, heart failure; HVPG, hepatic vein pressure gradient; LTx, liver transplantation; NASH, nonalcoholic steatohepatitis; S.C., subcutaneously; SGLT-2, sodium-glucose cotransporter-2.

#### Metformin

Metformin is largely accepted as first-line glucose-lowering treatment for T2D, although its position is challenged by novel antidiabetic drugs.^30^ Metformin reduces hepatic glucose production by suppressing gluconeogenesis, increases fatty acid oxidation, inhibits lipolysis and fatty acid release from adipose tissue, improves peripheral insulin sensitivity, and reduces intestinal glucose absorption^31^; however, it has no effects on NASH progression.^32^

The main risk associated with metformin use is decreased lactate conversion into pyruvate, with possible occurrence of lactic acidosis. This risk is fostered by renal failure,^33^ and chronic renal failure is common in nonrenal organ transplant, particularly in the presence of diabetes (relative risk [RR], 1.42; 95% confidence interval [CI], 1.33-1.51).^34,35^ Metformin-associated lactic acidosis is rare,^36^ but dosage must be tapered down and finally stopped in subjects with stage IV chronic kidney disease (eGFR <30 mL/min/1.73 m^2^),^37,38^ not in cirrhosis per se.^39^ This is also the case of patients who are likely to be rapidly transplanted (patients at the top of the waiting list) to avoid the risk of acute kidney injury and lactic acidosis associated with surgery, hypotension, and blood loss in the early posttransplant phase.^35^

In 250 patients with diabetes and cirrhosis of different severity, continuing use of metformin significantly reduced all-cause mortality, and longer survival extended to Child B/C patients, after adjusting for confounders (hazard ratio [HR], 0.43; 95% CI, 0.24-0.78).^40^ Metformin was also reported to reduce the incidence of hepatic encephalopathy in a cohort of 82 patients with cirrhosis and T2D, possibly via inhibition of glutaminase activity.^41^

Finally, metformin has an anticarcinogenic effect, confirmed in several cohort studies and on several cancer sites,^42^ including the liver. In a meta-analysis including 105 495 patients with T2D, metformin significantly reduced the risk of liver cancer (odds ratio [OR], 0.38; 95% CI, 0.24-0.59) and specifically of HCC (0.30; 0.17-0.52).^43^ In 100 patients with HCV-related cirrhosis and diabetes without contraindication for metformin use (median follow-up, 5.7 y), the drug reduced HCC occurrence (HR, 0.19; 95% CI, 0.04-0.79) and liver-related deaths or transplantation (0.22; 0.05-0.99).^44^ In conclusion, metformin should be maintained in patients with diabetes and cirrhosis, provided that renal function is frequently monitored and metformin is stopped in conditions favoring dehydration or tissue hypoxia.

#### Pioglitazone

Glitazones activate peroxisome proliferator-activated receptor-γ, a widely diffused nuclear receptor, mostly expressed in adipocytes. Activation of the peroxisome proliferator-activated receptor-γ reduces insulin resistance, promotes the differentiation of adipocytes, decreases leptin and interleukin (IL)-6, and increases adiponectin levels,^45^ inhibiting collagen synthesis from hepatic stellate cells.

Pioglitazone is the only glitazone on the market following the withdrawal of rosiglitazone for safety concerns in most countries regarding the CV system. According to official labeling, it should not be prescribed in case of liver disease or aminotransferase levels >2.5 times the upper normal limit.^39^ Nonetheless, in a meta-analysis of biopsy-confirmed NASH patients included in 5 randomized controlled trials (RCTs), pioglitazone was associated with a significant improvement of advanced fibrosis (OR, 4.53; 95% CI, 1.52-13.52), as well as with NASH resolution (3.51; 1.76-7.01),^46^ irrespective of diabetes. The effects were confirmed by the immediate flare-up of alanine aminotransferase after drug discontinuation, possibly heralding NASH recurrence.^47^ According to international guidelines, this makes pioglitazone the long-term treatment of choice for NASH, also useful in patients with cirrhosis of other cause, despite limitations and risks of adverse events. Its use is hindered by fluid retention, favoring heart failure^48^ and possibly ascites. This adds to increased body weight and to the risk of nonosteoporotic fractures.^48^

Notably, pioglitazone has beneficial effects also on coronary artery disease and the cerebrovascular system^49,50^ and reduces the risk of hepatocellular and colorectal cancer.^51,52^

#### Sulfonylureas and Glinides

Sulfonylureas stimulate insulin release from pancreatic β-cells in a glucose-independent manner, binding to ATP-sensitive potassium channels on pancreatic β-cells. In general, these drugs are metabolized by the liver, bind to serum proteins, and are excreted by the kidney.^53^ Glinides bind the same receptor as sulfonylureas, showing the same mechanism of action, and are similarly bound to albumin but have a shorter half-life (namely, repaglinide).

Considering their pharmacokinetics and mechanism of action, these drugs carry a high risk of hypoglycemia in the presence of decompensated cirrhosis because of increased plasma concentration favored by reduced metabolism and hypoalbuminemia,^54^ poor nutritional status, and, in patients with alcohol consumption inhibition of hepatic gluconeogenesis.^39^

Sulphonylureas/glinides are very effective on glucose levels, but, independently of liver disease, their use has been moved to third-line treatment in recent guidelines because novel drugs are safer, similarly effective, and not associated with the CV risk of insulin secretagogues.^55^ Their use should be even more discouraged in the presence of liver disease.

#### Acarbose

Alpha-glucosidase inhibitors lower postprandial glucose levels by reducing intestinal carbohydrate absorption. They have a modest activity on glucose control but are a valid and safe add-on treatment in pre-LTx cirrhosis, also in the more advanced stages, because of low systemic bioavailability and no hepatic metabolism.^56^ In a cross-over comparison (100 mg × 3 daily versus placebo for 8 wk) in T2D patients with cirrhosis and low-grade hepatic encephalopathy, acarbose significantly reduced fasting and postprandial glucose levels, improved ammonia and neuropsychological function.^57^ The effects on encephalopathy might be related to increased bowel movements without change in liver function.

#### DPP-4 Inhibitors (Gliptins)

Dipeptidyl peptidase-4 inhibitors (DPP-4Is) have been on the market since 2006. They act by inhibiting the degradation of glucagon-like peptide-1 (GLP-1) and glucose-dependent, insulinotropic polypeptide, responsible for insulin secretion by pancreatic β-cells and for suppression of glucagon.^58^ This mechanism implies a low risk of hypoglycemia because insulin secretion is progressively stopped by declining glucose.^59^ All DPP-4Is are metabolized in the liver and excreted by the kidneys, except for linagliptin, undergoing biliary excretion.^60^ Although mild pharmacokinetic changes are observed in relation to declining liver function, these compounds do not require dosage adjustment.^61^ Only for vildagliptin, the product characteristics do not suggest administration in patients with liver disease or pretreatment alanine aminotransferase or aspartate aminotransferase >3-fold increased.

Glucose control is variable between gliptins; the average reduction of HbA1c ranges between 0.5% and 0.8% with monotherapy, with very rare and mild side effects limited to flu-like symptoms. All gliptins, except vildagliptin, proved noninferior but not superior to standard treatment in cardiovascular outcome trials (CVOTs), as recommended by the United States Food and Drug Administration.^62^ The evidence was reinforced by meta-analyses, also showing their inferiority versus glucagon-like peptide-1 receptor agonists (GLP-1RAs) and sodium-glucose cotransporter-2 inhibitors (SGLT-2Is) on CV, renal, and heart failure outcomes.^63,64^ Limited data exist in cirrhosis, including patients with NASH cirrhosis, but their use may be justified considering safety and easiness of use (oral route, once-daily administration), provided that a progressive dose adjustment is considered in kidney disease (up to end-stage and dialysis).^65^

#### GLP-1 Receptor Agonists

GLP-1RAs represent an attractive therapeutic option for T2D treatment in cirrhosis. GLP-1 is a gut-derived incretin that stimulates insulin release and suppresses glucagon secretion, inhibits gastric emptying, and reduces appetite and food intake. GLP-1RAs are synthetic molecules similar to endogenous GLP-1 but resistant to dipeptidyl peptidase-4 (DPP-4) degradation.^66^ Direct and indirect comparisons of daily or weekly subcutaneous GLP-1 injections with other agents showed that GLP-1RAs are the most potent antihyperglycemic drugs available at present. Semaglutide is also approved for oral daily administration.

Safety data for GLP-1RAs in liver disease only come from case reports or retrospective studies in T2D patients with elevated liver enzymes or NASH.^67,68^ Considering their renal elimination, no dose adjustment is advisable for lixisenatide and exenatide, as well as for dulaglutide, according to its kinetic properties.^69^ As to liraglutide, a kinetic study reported lower levels with liver dysfunction, suggesting differences in absorption from the subcutaneous tissue or decreased albumin binding.^70^

An anecdotical case supports the use of liraglutide in T2D patients with cryptogenic cirrhosis; more data are derived from liraglutide studies in NASH with or without fibrosis.^71^ Liraglutide dose-dependently reduced liver enzymes, possibly as a consequence of reduced body weight and liver fat.^71^ In the Liraglutide Efficacy and Action in NASH study in biopsy-proven NASH, also including patients with advanced fibrosis and cirrhosis (50% of cases), liraglutide led to 35% NASH resolution (versus 8% in placebo; OR, 7.8; 95% CI, 1.3-46.7) but no significant effect on fibrosis.^72^ Similar data were recently reported in a phase 2b RCT of daily semaglutide. NASH resolution without fibrosis worsening was significantly attained versus placebo at all tested doses, with a maximum (OR, 6.87; 95% CI, 2.60-17.63) at 0.4 mg/d, a much higher dose than commonly used in diabetes (1.0 mg/wk). However, the confirmatory end-point of fibrosis improvement without NASH worsening was not reached.^73^ In real-world studies and in CVOTs, GLP-1RAs use has been associated with improved steatosis and fibrosis biomarkers,^74-77^ and similar data have been reported in a study measuring the clinical effects of switching from metformin with or without sulfonylureas to GLP-1RAs,^78^ but histologic data are limited.

Several additional effects of GLP-1RA should be considered. In keeping with their properties on gastric emptying and central nervous system, treatment is associated with nausea, low appetite, and abdominal discomfort, leading to important weight loss, which may further be enhanced by lifestyle intervention.^79^ The weight loss effects of high-dose semaglutide (2.4 mg/wk) are by far the largest ever attained by drug treatment in obesity,^80^ useful in patients with metabolic syndrome. However, nausea may negatively affect patients with poor nutritional status and is a major cause of drug discontinuation. CVOTs have consistently shown a decreased risk of CV events; a comprehensive meta-analysis estimated a significant overall reduction of major adverse CV outcomes) (HR, 0.88; 95% CI, 0.82-0.94), without heterogeneity across individual outcomes.^81^ All-cause mortality was reduced by 12%, hospital admission for heart failure by 9%, and a composite kidney outcome by 17% (HR, 0.83; 95% CI, 0.78-0.89).

A matter of concern comes from a case series of 18 patients, simultaneously treated with liraglutide and propranolol for diabetes and cirrhosis at risk of bleeding. The expected heart rate target of 55–65 beats/min (surrogate marker of efficacy for bleeding prevention) was not achieved under liraglutide, despite up-titration of propranolol.^82^ A modest increase in heart rate is a well-known effect of GLP-1RAs, negligible in the general population, but studies are needed in this setting.

In conclusion, because of their general safety, efficacy on metabolic control, and positive results in liver disease and CV protection, GLP-1RAs are recommended in cirrhosis, with few limitations.

#### SGLT-2 Inhibitors (Gliflozins)

SGLT-2Is block a transporter present almost exclusively in the proximal renal tubules and responsible for nearly 90% reuptake of glucose filtered at glomerular level. By favoring glucose excretion in the urines, SGLT-2Is reduce blood glucose levels by an insulin-independent mechanism, with nearly null risk of hypoglycemia.

The most common SGLT-2Is (canagliflozin, dapagliflozin, and empagliflozin) are metabolized in the liver, but no dosage adjustment is required in mild or moderate (for canagliflozin and dapagliflozin) or even severe liver failure (empagliflozin).^83-85^ Because canagliflozin exposure is also associated with creatinine clearance, caution is mandatory in combined hepatic and renal impairment.^83^ SGLT-2Is reduce the renin-angiotensin-aldosterone-system activity and might exert a potential role in preventing and treating ascites in patients with cirrhosis,^86^ but low renal function limits their effectiveness.

Gliflozins have been tested in real-world and RCT studies for their effects on biomarkers of steatosis and fibrosis with positive results,^87-89^ but very few histologic data are available. A reduction in liver enzymes is commonly observed during treatment, larger when compared with other antidiabetic drugs. Two biomarkers of steatosis and fibrosis (Fatty liver Index^90^ and fibrosis-4 score^91^) significantly improved after switching from old antidiabetic agents to SGLT-2Is in 195 T2D patients.^78^ Also in this case the effect might be partly explained by weight loss, comparable to that observed following GLP-1RA initiation. In a network meta-analysis, gliflozin treatment was associated with weight loss ≥5% versus placebo (dapagliflozin: OR, 8.57; 95% CI, 2.71-27.44; empagliflozin: 10.20; 4.59-28.93).^92^

Notably, CVOTs and epidemiological studies showed that also gliflozins are associated with improved CV—particularly heart failure—and renal outcomes. A meta-analysis of 63 studies concluded for protective effects of SGLT-2Is against the risk of composite major adverse CV outcomes (RR, 0.84; 95% CI, 0.75-0.95), to CV death (0.63; 0.51-0.77), heart failure (0.65; 0.50-0.85), and all-cause death (0.71; 0.61-0.83), not for nonfatal myocardial infarction or angina.^93^ Beneficial effects were later shown on renal outcomes, from delayed albuminuria progression (RR, 0.71; 95% CI, 0.66-0.76) to albuminuria regression (1.71; 1.54-1.90), a combined renal outcome (0.57; 0.49-0.66), and all-cause mortality (0.84; 0.75-0.94).^94^ Similar effects are also present in heart failure patients without diabetes,^95^ a totally new area of research.^96^

Genital infections are the principal adverse events of gliflozins, as derived from spontaneous reporting systems^97^ and from an extensive meta-analysis (RR, 4.19; 95% CI, 3.45-5.09)^98^; warnings about diabetic ketoacidosis have also been raised.^99^ No specific data are available in cirrhosis, a population at increased risk of infection, where close monitoring of electrolytes and renal function and careful education to prevent infections should accompany their use.

#### Insulin and Insulin Analogs

Insulin treatment remains the final choice to obtain metabolic control. Insulin requirements are very high in ESLD, characterized by insulin resistance, despite low insulin clearance due to portosystemic shunting.^100^ In Western countries, insulin analogs are almost universally used; they provide better glucose control compared with human insulin, although at higher cost. The metabolism of both short-acting (aspart, lispro, glulisine) and long-acting (glargine, detemir, degludec) analogs has not been systematically studied in cirrhosis. Reduced renal clearance and defects in renal gluconeogenesis are present in chronic kidney disease,^101^ and dose adjustment of insulin is needed (up to 50% dose reduction for eGFR <10 mL/min/1.73 m^2^).^102^

Insulin use carries a high risk of hypoglycemia, also favored by portosystemic shunting; in cases treated by β-blockers, the risk is complicated by hypoglycemia unawareness, increasing the likelihood of brain damage. In addition, the need for glucose monitoring heavily affects health-related quality of life^103^ and adherence^104^ in patients with multimorbidity and complex regimens.^105^ Weight gain is an additional problem. Both risks make the sole use of basal insulin preferred versus basal-bolus treatment, provided that metabolic control is achieved. A final problem is the well-known association of insulin with cancer risk,^106^ including HCC. In a meta-analysis of 5 cohort and 9 case–control studies, insulin treatment was associated with a RR for HCC of 1.90 (95% CI, 1.44-2.50) versus noninsulin users.^107^ The potential bias of longer follow-up was not excluded.

In conclusion, insulin use should be avoided as long as possible in advanced liver disease. The combined use of basal insulin and GLP-1RAs might be a suitable therapeutic option.^108^

#### Intensive Lifestyle Intervention

Intensive lifestyle intervention (ILI) is the backbone of NAFLD treatment, extensively proven in real-world observational studies. ILI strategies, based on the principles of the Diabetes Prevention Program,^109^ aimed at 7%–10% weight loss targets^110^ are known to reduce fibrosis biomarkers^111^ and to improve liver histology,^110,112^ as well as cardiometabolic risk factors.^113^ Similar results were also reported in cirrhosis and should be implemented in patients before and after waitlisting. In a prospective, uncontrolled study, overweight patients with compensated cirrhosis and portal hypertension enrolled in a 16-wk ILI program significantly reduced hepatic vein pressure gradient (by ≥10% in 42% of the cases and ≥20% in 24%) and body weight (by ≥5% in half of the cases and ≥10% in 16%).^114^ The program included personalized diet and supervised physical activity (60 min/wk), provided by a dedicated team rarely present in liver units, and continuing patient–team interaction. This last condition may be partly overcome by e-technology coupled with web-based programs, similarly effective in motivated patients.^115^

One study directly compared ILI versus liraglutide (3 mg/d) in diabetes; in the short term, no differences were observed in weight loss, biochemistry, and measures of fibrosis,^116^ but weight loss maintenance was sustained in ILI, not after liraglutide stop.^117^

#### Bariatric Surgery

Evidence supporting bariatric surgery is exclusively derived from observational studies: Roux-en-Y-gastric bypass and sleeve gastrectomy—the surgical procedures of choice^118,119^—are associated with both fibrosis regression^119^ and reduced progression to cirrhosis (HR, 0.31; 95% CI, 0.19-0.52).^120^ The beneficial effect on diabetes reversal is large^121^ and sustained^122^ and adds to the reduced incidence of cancer and CV events in surgically treated obesity.^123,124^ Cirrhosis per se is not an absolute contraindication to bariatric surgery, but mortality risk increases systematically with decreased liver function (compensated cirrhosis: OR, 2.17; 95% CI, 1.03-4.55; decompensated: 21.2; 5.39-82.9).^125^ Accordingly, bariatric surgery is contraindicated in patients with decompensated cirrhosis, moderate or severe thrombocytopenia, malnutrition, and sarcopenia, and only a minority of LTx candidates can be considered for sleeve gastrectomy, by far the most common surgical technique.^126^ Decisions should be based on hepatic functional reserve, portal hypertension, and CV risk^127^; additional factors to consider are type and timing of bariatric surgery, the potential perioperative/postoperative complications of portal hypertension, and the possible impact on the absorption of immunosuppressive therapy.

Surgery long before LTx, by reducing comorbidity progression, is likely to facilitate candidacy in subjects with severe obesity^128^; on the other hand, any bariatric surgery-related complication might increase the risk of delisting or delay LTx.^129^ In a recent retrospective single-center study, sleeve gastrectomy in 14 eligible waitlisted patients resulted in sustained weight loss and significantly lower risk of diabetes (OR, 0.04; 95% CI, 0.00-0.41), hypertension (0.15; 0.04-0.67), and recurrent or de novo NAFLD post-LTx (HR, 0.19; 95% CI, 0.04-0.91) versus 56 eligible controls undergoing medical weight loss.^130^ Combined sleeve gastrectomy and LTx is an alternative option, but very few cases have been published.^131,132^ This option is burdened by risks associated with unexpected LTx difficulties and by post-LTx nutritional problems. Bariatric surgery after liver transplantation is also challenging because of patients’ comorbidities and technical problems such as adhesion, bleeding, and surgical site infection related to prior abdominal surgery, but the overall effects are not different from those observed in non-LTx subjects with obesity.^133^ Only hospital stay was longer (3.1 versus 1.7 d in non-LTx; *P* < 0.001), but the procedure did not require correction of immunosuppression medication needs.^133^

Also, mortality and graft survival should be considered. In a meta-analysis of 187 patients submitted to bariatric surgery before LTx (8 studies), no 30-d mortality was recorded and 1-y graft survival was 70%. Combined LTx and bariatric surgery in 32 cases (2 studies) resulted in no 30-d mortality and 100% graft survival. Post-LTx surgery (64 cases) was followed by no 30-d mortality but 7.8% mortality within 1 y.^134^ Overall, evidence favors post-LTx surgery, with 45% rates of diabetes regression.^135^

In conclusion, bariatric surgery may be feasible either as a bridge to LTx (to favor candidacy) or post-LTx to reduce comorbidities. A multidisciplinary team is mandatory to identify optimal timing, type of surgery, and to secure long-term metabolic monitoring.

### Prevalence of and Risk Factors for Posttransplantation Diabetes

T2D is also common among transplanted patients, but the pathogenesis differs in relation to pre-LTx glucose metabolism. In subjects with pre-LTx diabetes, the condition may either persist or resolve following transplantation, largely modulated by β-cell dysfunction^23^; in other cases, new-onset PTDM may develop. The condition must be verified after reaching clinical stability; it is important to split the post-LTx period into early and late phases, considering the possible presence of stress-related hyperglycemia in the early postsurgical period.^136^

#### Early Stress-related Hyperglycemia

In the early, postoperative phase, hyperglycemia is the rule. Defective glucose metabolism stems from the hormonal and cytokine storm of surgery and possible infections,^137^ favored by high-dose steroid therapy at surgery and tapered doses thereafter, and both peripheral and hepatic insulin resistance.^138^ Mild-to-moderate stress hyperglycemia is protective, providing fuel for the immune system and the brain to cope with higher needs; however, persistent hyperglycemia becomes deleterious,^139^ contributing to adverse outcomes via free radicals (oxidative stress), endothelial dysfunction, inflammatory responses, and vascular dysfunction.^140^ During this phase, insulin treatment is the rule and should be provided by infusion, as detailed by guidelines,^141^ although most protocols lack a robust evidence base. As soon as possible, preplanned basal-bolus regimens should be started; they provide more accurate control and reduce the risk of hypoglycemia compared with sliding-scale programs.^142^

#### Late Hyperglycemia

**FIGURE 3.**
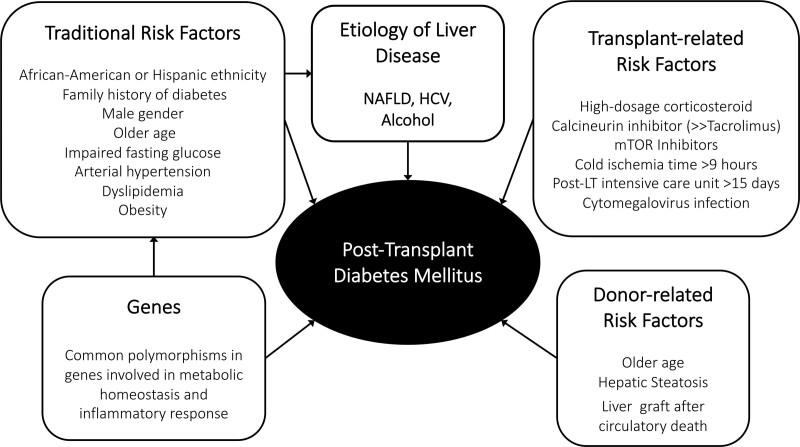
Risk factors associated with development of PTDM in patients with liver disease. Note that both genes and traditional risk factors also contribute to PTDM via development and progression of NAFLD. HCV, hepatitis C virus; LT, liver transplant; mTOR, mammalian target of rapamycin; NAFLD, nonalcoholic fatty liver disease; PTDM, posttransplantation diabetes mellitus.

PTDM can be diagnosed only if hyperglycemia (again, fasting glucose ≥126 mg/dL or random glucose ≥200 mg/dL) persists for at least 45 d after LTx.^2^ PTDM is a multicausal disease, adding immunosuppressive drug use and donor factors^138,143,144^ to background characteristics shared with pre-LTx T2D (Figure 3), that is, dysfunctional insulin release and an impaired gut–pancreas incretin axis, as well as abdominal obesity, whenever present.

Different studies from Western and Asian countries reported a prevalence of PTDM ranging from 8.4% to 40%^138,145-152^; this wide variability depends on the diagnostic criteria, risk factors for de novo diabetes, and baseline characteristics. As for general population, risk factors are Hispanic or African American ethnicity (1.6-fold increased risk),^150^ male gender, older age, familiarity for diabetes, and metabolic risk factors (perisurgical blood glucose,^148^ arterial hypertension,^153^ and higher preoperative triglycerides, even within a near-normal range).^154^ A recent meta-analysis pooling data from 37 studies showed that pre-LTx body mass index and overweight were associated with a higher risk of PTDM, while no association was found with post-LTx body mass index.^155^ Notably, PTDM prevalence increases progressively in the posttransplant period, and in a single-center study of 415 adult LTx recipients, it was 34.7%, 46.9%, and 56.2% at 1, 3, and 5 y, respectively, with older age remaining as the only independent predictor.^156^

### Specific Risk Factors for PTDM

#### Cause of Underlying Liver Disease

Large cohort studies in LTx recipients showed that the risk of PTDM was higher in liver disease of NAFLD/NASH^150,152^ or HCV infection^150^ cause. Among 35 870 LTx recipients from 1994 to 2013 who reached 5-y follow-up, pre-LTx T2D was present in 11.2% and PTDM in 29.7% after a mean period of 76 mo.^147^ Patients transplanted for NASH had an increased risk of PTDM at any time after LTx, even after the first year, when the diabetogenic effect of immunosuppression therapy should be reduced.^157^ More recent studies did not confirm the association between NASH and PTDM. In 2019, Lieber et al^156^ confirmed the rapid onset of PTDM in 34.9% of patients in the first year post-LTx; at multivariate analysis, age was the only independent risk factor for PTDM, while NASH was associated with a higher pre-LTx DM rate, not with increased PTDM. The growing pandemic of NAFLD and of its comorbidities^158,159^ will definitely increase the future burden of NASH-associated PTDM.

As to HCV infection, in a meta-analysis of 7 studies including 1899 LTx recipients, HCV increased the risk of PTDM (OR, 2.46; 95% CI, 1.44-4.19) versus non-HCV LTs in the immediate posttransplant period and at longer follow-up (1.39; 1.06-1.83).^160^ DAA-based therapies for HCV infection reduced the overall burden of HCV and the number of viremic transplanted patients in the last years,^26,27^ and this predicts the decline of PTDM in future HCV series.^161-163^ Finally, among preexisting or acquired viral infections, the role of cytomegalovirus in PTDM has not been clarified. Data from China Transplant Registry in non-DM LTx recipients showed an association of cytomegalovirus infection with a higher risk of PTDM,^164^ but conflicting results on kidney and liver transplantation are also available.^165-168^

#### Immunosuppressive Therapy

Steroids are primarily used during the first 6 mo and long term, maintained in recipients transplanted for autoimmune disorders or recurrent rejection episodes, that is, longer than the classical 45 d set in the definition of PTDM. Glucocorticoids induce peripheral and hepatic insulin resistance, with increased hepatic glucose output and reduced peripheral metabolism in adipose and skeletal muscle tissues, with a peak effect after 4–8 h and a duration of 12–16 h.^169^ The effects are maintained during long-term low-dose prednisolone therapy.^170^

Calcineurin inhibitors (CNIs—ciclosporin and tacrolimus) are the backbone of maintenance immunosuppressive therapy in solid organ transplantation; tacrolimus is the most frequently used drug. Their diabetogenic effects may be explained by downregulation of β-cell proliferation, insulin production, and insulin release; a recent meta-analysis confirmed that ciclosporin is less diabetogenic versus tacrolimus (OR, 0.60; 95% CI, 0.47-0.77),^171^ consistent with an older meta-analysis indicating a dose-dependent risk associated with tacrolimus (RR, 1.86; 95% CI, 1.11-3.09)^172^ and with a large single-center retrospective analysis in LTx patients (tacrolimus: OR, 2.76; 95% CI, 1.78-4.30; sirolimus: 2.72; 1.67-4.42).^152^ These diabetogenic risks are even larger with the inhibitors of mammalian target of rapamycin (mTOR)(everolimus and sirolimus) that contribute to downregulation of gene expression for insulin release, β-cell apoptosis, and peripheral insulin resistance.^138,173-175^ The biochemical mechanism might be an interaction with Mg^+^ transport, favoring excretion in the urines and hypomagnesemia.^176,177^ Low blood magnesium, mainly in the first post-LTx month,^178^ is a risk factor for PTDM, independent of the immunosuppressive regimen.^176^

Mycophenolate mofetil, a reversible inhibitor of inosine monophosphate dehydrogenase, not only exerts a lower immunosuppressive activity compared with CNIs but also carries a lower risk of PTDM.^179^ It is added as a double or triple regimen to CNIs and steroids to mitigate their diabetogenic effects,^180,181^ and this strategy may successfully control glucose metabolism. Also, azathioprine, now largely replaced by mycophenolate mofetil in immunosuppressive protocols, has no significant impact on PTDM.

Basiliximab, a humanized monoclonal antibody against IL-2 receptor, is the only lymphocyte nondepleting agent used as an induction agent in steroid-sparing or CNI minimization regimens particularly in pediatric LTx.^182^ A meta-analysis of 6 studies in steroid-free immunosuppressive regimens between 1998 and 2015 showed that its use reduced PTDM risk (RR, 0.56; 95% CI, 0.34-0.91).^183^ In a recent RCT, basiliximab induction, followed by tacrolimus plus azathioprine maintenance, significantly reduced the incidence of PTDM versus steroid induction both at 3 (64.5% versus 28.1%) and at 6 mo (51.6% versus 15.6%).^184^

#### Liver Donor-related Factors

The growing gap between the need for and the supply of transplantable organs has extended the criteria for donor livers. This led to extended donor age and donor-related comorbidities, such as diabetes, hypertension, or liver steatosis, predisposing to PTMD as well as older liver donor graft after circulatory death.^144,164,185-187^

#### Genes

Common polymorphisms in recipient genes involved in metabolic homeostasis and inflammatory response—SUMO4, ADIPOQ, Angiotensinogen, STAT4, IL-18, mTOR—have been associated with an increased risk of PTDM, but evidence arises mostly from small Asian studies and needs further validation.^188-192^

### Impact of Diabetes on Posttransplant Outcomes

Diabetes per se affects the quality of life of LTx patients and contributes to complications, drug–drug interactions, and disease burden, but the association of PTDM with adverse events and outcomes remains controversial (Table 4). With the sole exception of a Taiwan database,^149^ where the incidence of PTDM, exclusively based on International Classification of Diseases, Ninth Revision, Clinical Modification codes, might be severely underdiagnosed, there is a general consensus for an overall risk of death in PTDM at least comparable with that observed in pre-LTx diabetes. However, although overall diabetes and PTDM were both risk factors of mortality in the SRTR study,^151^ only pre-LTx diabetes emerged as an independent risk factor of CV mortality, accounting for approximately 10% of all transplant deaths.^147^ Similarly, in the Toronto experience, no differences in survival were demonstrated between patients with/without diabetes, either pre- or post-LTx.^152^ In this analysis, the risk for CV events and end-stage renal disease associated with PTDM was intermediate between pre-LTx- and no-diabetes.^152^ In the old National Institute of Diabetes and Digestive and Kidney study, both pre-LTx and PTDM were predictors for 1-y mortality at univariate analysis. When pre-LTx patients with diabetes were excluded, PTDM (excluding transient diabetes) maintained a significant relation with long-term death risk, but only pre-LTx diabetes predicted 5-y death at multivariate analysis.^146^ Finally, in a retrospective cohort study, PTDM patients had a 3-fold higher incidence of major CV events versus no-DM patients (18.7 versus 5.5%) and, in patients with events, the risk of death was 3-fold higher.^150^ A specific association between diabetes and the risk of renal failure was also reported on the basis of a combined analysis of 69 321 transplants of nonrenal organs (liver, heart, lung, heart-lung, and intestine) in the period 1990–2000.^35^

**TABLE 4.** Principal and most recent studies investigating the association of PTDM with main outcomes

**Author, y**	**No. of cases follow-up**	**All-cause death**	**CV mortality or events**	**Liver-related mortality acute rejection or re-LTx**	**End-stage renal disease**
Watt (NIDDKD database), 2010^146^	N = 798Median, 10 y	Not significant; Pre-LTx DM, HR 1.48 (1.06-2.08)	Pre- and post-LTx DM not significant	HR 1.85 (1.21-3.05) for pre-/post-LTx DM	—
Younossy (STRT registry), 2014^147^	N = 85 194Mean, 6.5 y	HR 1.06 (1.02-1.11); Overall DM, HR 1.21 (1.12-1.30)	Not significant; Mortality in Pre-LTx DM, HR 1.93 (1.55-2.41)	—	—
Liu (Taiwan Health Insurance database), 2016^149^	N = 2248Up to 14.5 y	Higher survival rates vs pre-LTx DM and no-DM (*P* = 0.021)	—	—	—
Roccaro (UPHS database), 2018^150^	N = 994Median, 54.7 mo	HR 1.61 (1.05-2.48) at multivariable analysis	HR 1.81 (1.14-2.90); no longer significant at multivariable analysis	HR 1.92 (1.35-2.72) at multivariable analysis	HR 2.06 (1.56-2.72); not significant at multivariable analysis
Bhat (STRT registry), 2018^151^	N = 61 697Data at 10 y	HR 1.55 (1.50-1.59), similar to pre-LTx DM (1.54; 1.46-1.61)	—	—	—
Aravintham, 2019^152^	N = 2209Median, 6.7 y	No differences in survival vs no-DM and pre-LTx DM	Risk similar to pre-LTx DM, in turn higher vs no-DM (*P* < 0.01)	No differences in ESLD requiring re-LTx vs no-DM and pre-LTx DM	Risk similar to pre-LTx DM, in turn higher vs no-DM (*P* = 0.003)

HRs (95% confidence intervals) refer to PTDM vs no-DM if not otherwise specified. All values are adjusted for confounders.

CV, cardiovascular; DM, diabetes mellitus; ELSD, end-stage liver disease; HR, hazard ratio; LTx, liver transplantation; NIDDKD, National Institute of Diabetes and Digestive and Kidney; PTDM, posttransplantation diabetes mellitus; STRT, Scientific Registry of Transplant Recipients; UPHS, University of Pennsylvania Health System.

In conclusion, the impact of PTDM might be considered within the complex picture of LTx patients, where preexisting conditions long impairing the liver, the kidneys, and the CV system, together with the new metabolic conditions associated with altered glucose metabolism, are very likely to produce negative effects on long-term outcomes.

### PTDM Treatment

In general, PTDM treatment does not systematically differ from the treatment of any patient with diabetes. Frailty suggests a conservative approach until patients reach a stable condition and justifies an intense metabolic control following LTx, with treatment aimed at HbA1c targets not at risk of hypoglycemia.

**FIGURE 4.**
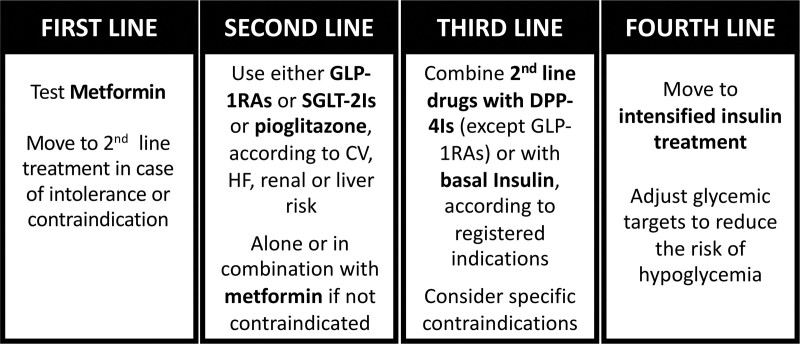
Treatment intensification with glucose-lowering drugs in pre- and post-LTx patients with diabetes. Intensification depends on glucose targets and patients’ frailty. CV, cardiovascular; DPP-4I, dipeptidyl peptidase-4 inhibitor; GLP-1RA, glucagon-like peptide-1 receptor agonist; HF, heart failure; LTx, liver transplantation; SGLT-2I, sodium-glucose cotransporter-2 inhibitor.

In stable post-LTx patients, pharmacologic treatment should follow the international guidelines, suggesting a progressive implementation of different antidiabetic agents (Figure 4).^30^ The most compelling issues are the frequent changes in drug schedule, drug–drug interactions, and immunosuppressive treatment. Pharmacokinetic studies indicate a low risk of interaction between the different classes of antidiabetic drugs and CNIs or mTOR inhibitors.^193^ However, metformin and acarbose may cause diarrhea and the accelerated intestinal transit may be responsible for malabsorption of immunosuppressive drugs, resulting in erratic blood levels, with graft risk. Accordingly, metformin must be slowly increased to optimal dosage in new-onset PTDM, slow-release formulations are preferable, and acarbose should not be used.

Adherence to lifestyle recommendations is mandatory to reduce CV risk and to control PTDM. Successful transplantation is frequently followed by rapid weight gain, abdominal obesity (up to 40%),^194^ and PTDM.^195^ In the long term, adherence to healthy lifestyle declines, driven by recovered well-being. ILI programs have been developed to reduce nonadherence^196^ and to prevent graft steatosis.^197^ They are more effective than pioglitazone to treat de novo allograft steatosis.^198^ Electronic health lifestyle programs, supported by dietitians or lifestyle trainers, have been designed to increase acceptability, tailored to recipients’ needs.^199^

The importance of bariatric (metabolic) surgery in this phase has previously been discussed.

## CONCLUSIONS

Diabetes remains an important comorbidity throughout the whole clinical life of patients with liver disease, affecting quality of life, direct and indirect costs, and finally outcomes. In the LTx setting, both in waitlisted patients and after transplant, several key messages should be conveyed (Table 5); they form a decalogue that reflects the evidence derived from the literature and hints from personal experience. The area is receiving a lot of attention, driven by the increasing number of NASH-associated candidates, the many new glucose-lowering drugs on the market requiring definition of the appropriate timing and sequence, and progress in bariatric surgery, which will ultimately resolve diabetes in selected cases. This clinical area of research is likely to expand further in the near future, fueled by clinical and commercial interests.

**TABLE 5.** A decalogue with key messages for the treatment of diabetes in the setting of liver transplant

Key messages
• Support healthy diet and moderate physical activity through continuous counseling by dietitians and lifestyle trainers, according to liver disease cause and severity, timing to surgery, and comorbidities.
• Provide intensive programs of cognitive behavior therapy delivered by dedicated teams to support weight loss or limit the negative effects of sarcopenia in waitlisted patients with obesity, as well as to prevent unhealthy post-LTx weight gain.
• Define HbA1c target considering patients’ frailty and the risk of hypoglycemia and CV events, which significantly affect all-cause, CV, and renal outcomes. Consider that HbA1c might not reflect metabolic control in anemia or recent bleeding.
• Maintain tapered metformin administration in waitlisted patients, except for eGFR <30 mL/min (CKD stage ≥4). Scale up metformin or use slow-release formulations in PTDM to avoid GI symptoms and interactions with immunosuppressive drug absorption.
• Use GLP-1RAs and SGLT-2Is with confidence unless contraindicated by severe CKD.
• Consider the possible interaction of GLP-1RAs with β-blocking agents in the prevention of GI bleeding in cirrhosis. Check electrolytes and blood pressure in patients treated with SGLT-2Is and taper down Henle loop diuretics.
• Treat PTDM according to schedules as simple as possible, limiting polypharmacology to avoid drug–drug interactions and to favor adherence.
• Limit the use of insulin to the sole basal insulin as long as possible, both in waitlisted and in post-LTx patients, to reduce the risk of hypoglycemia and sustain quality of life. The association of basal insulin and GLP-1RAs may be a feasible alternative to intensified insulin treatment.
• Modulate immunosuppressive therapy, using steroid-free regimens with basiliximab induction or adding mycophenolate mofetil to mitigate the diabetogenic effects of calcineurin inhibitors (particularly tacrolimus). Do not use acarbose in the post-LTx phase to avoid diarrhea and malabsorption.
• Consider bariatric surgery to treat diabetes in selected cases, particularly in the post-LTx phase when associated with important weight gain.

CKD, chronic kidney disease; CV, cardiovascular; DM, diabetes mellitus; eGFR, estimated glomerular filtration rate; GI, gastrointestinal; GLP-1RA, glucagon-like peptide-1 receptor agonist; HbA1c, A1c glycated hemoglobin; LTx, liver transplantation; PTDM, posttransplantation diabetes mellitus; SGLT-2I, sodium-glucose cotransporter-2 inhibitor.
